# Potential prognostic markers of retained placenta in dairy cows identified by plasma metabolomics coupled with clinical laboratory indicators

**DOI:** 10.1080/01652176.2022.2145619

**Published:** 2022-11-13

**Authors:** Yuqiong Li, Huiyu Wen, Yuwei Yang, Zhengwei Zhao, Haihui Gao, Hongbing Li, Meizhou Huang

**Affiliations:** a Laboratory Institute of Animal Science, Ningxia Academy of Agricultural and Forestry Sciences, Yinchuan, China; b Academician (Expert) Workstation of Sichuan Province, The Affiliated Hospital of Southwest Medical University, Luzhou, China

**Keywords:** Cow, retained placenta, metabolomics, prognostic markers, liquid chromatography–mass spectrometry, hormone

## Abstract

The complex etiopathology of retained placenta (RP) and hazards associated with it has made it crucial for researchers and clinical veterinarians to study pathogenesis, early-warning diagnosis, and treatment. This study aimed to screen the potential prognostic markers of RP in dairy cows using plasma metabolomics coupled with clinical laboratory indicators. Blood samples were collected from 260 dairy cows at 21, 14, 7, and 0 days before parturition and 7, 14, and 21 days after parturition. Consequently, 10 healthy cows and 10 cows with RP with similar parity, body condition score, and age were included in the study. The changes in clinical laboratory indicators of the enrolled cows from 21 before parturition to 21 days after parturition were assessed. After initial overview of the multivariate statistical data using PCA analysis, the data were subjected to orthogonal partial least-squares discriminant analysis. Compared with cows with RP at 7 days before parturition, the levels of endothelin and 6-keto-prostaglandin F1α were increased in healthy cows, while the level of estradiol and progesterone decreased. Adenine dinucleotide phosphate, hypoxanthine, guanine dinucleotide phosphate, inosine monophosphate, and L-arginine were revealed as potential prognostic markers of cows with RP at 7 days before parturition involved in the regulation of taste transduction, purine and glutathione metabolism, and autophagy. The best period for the early-warning diagnosis of RP in dairy cows is 7 days before parturition, and purine metabolism and autophagy may play a vital role in the occurrence and development of RP in dairy cows.

## 1. Introduction

Retained placenta (RP), a prevalent complication in animals after parturition, increases the risk of uterine infections and infertility, increases calving interval, and reduces milk yield, causing great adverse efficiency for reproductive and productive performance in dairy cows (Qu et al. 2014; Gohary and LeBlanc 2018; Mahnani et al. 2021a). Moreover, RP could increase the delay in uterine involution, ovarian cystic degeneration, chronic endometritis, and pyometra. RP is the failure to expulse all or part of the placenta or fetal membranes within 24 h of calving (Qu et al. 2014; Peltoniemi et al. 2015). The complicated etiology of RP is affected by the management of environmental causes in herds and the physiological and pathological states of cows, including age, parity, heredity, hormones, and nutrition, and the condition of the calves, such as stillbirth and twinning (Mahnani et al. 2021b). These complex risk factors of RP pose a huge challenge in the prediagnosis and prevention of RP. There is no consensus on the treatment of RP, although systemic administration of antibiotics could effectively relieve RP complications, including metritis and pyometra (Beagley et al. 2010; Warnakulasooriya et al. 2018; Patrick et al. 2020). In addition, RP occurrence, despite effective treatment, considerably increases the risk of production and reproductive dysfunction in dairy cows (Risco and Hernandez 2003; Mahnani et al. 2021a). Therefore, early-warning diagnosis and early prevention have gradually become important means to effectively manage RP in dairy cows and alleviate losses associated with RP.

Placental maturation and separation are complicated biological processes involved in the reproductive, endocrine, and immune systems (Attupuram et al. 2016). Various biofactors, including cytokines, immune-inflammatory factors, and hormones, dynamically decreased with the spatiotemporal progression of placental maturation and separation (Boro et al. 2015; Dervishi et al. 2016; Shimizu et al. 2018; Khudhair et al. 2021; Ramos et al. 2022). Many studies have shown that alterations in biochemical and metabolite profiles, cytokines, and hormones could be screened as potential diagnostic biomarkers to track the biological RP process (Yazlık et al. 2019; Lu et al. 2020b). However, sensitive, stable, and early prediagnostic markers have not been identified yet.

The development of metabolomics technology has become a convenient and effective technical means to assist in the identification of biomarkers for disease prevention, diagnosis, and treatment (Amin and Hussein 2022). Many studies have confirmed that complex clinical phenotypes could be distinguished using several relevant biomarkers screened by employing targeted and untargeted metabolomics technology (Dervishi et al. 2018; Li et al. 2021; Zhang et al. 2021). The physiological and pathological processes of placental maturation and separation can also be dynamically monitored at the metabolomic level. Differential metabolites screened using metabolomics are susceptible to confounding factors. Therefore, plasma metabolomics coupled with the analysis of clinical laboratory indicators to explore the potential prognostic markers of RP in dairy cows helping to rule out confounding factors.

Because placental maturation and separation is a dynamic biological process, the sensitivity, specificity, and stability of the potential prognostic markers of RP depend on the selection of the detection period. Herein, a single-center, prospective study was conducted in a large-scale pasture, and plasma metabolomics coupled with clinical laboratory indicators was used to assess the potential prognostic RP markers. The balance in reproductive hormones is essential for the separation and expulsion of the placenta, whereas lower estradiol and endothelin levels may reduce uterine contractility, causing failure in expelling fetal membranes (Wischral et al. 2001a; Wischral et al. 2001b; McNaughton and Murray 2009). Moreover, increased MDA levels with decreased GSH-Px and SOD activities were found in the serum of postnatal cows with RP. Therefore, the clinical laboratory indicators of the cows included in this study were assessed in the transition period to determine the fluctuation in reproductive hormones, ET, and oxidation and antioxidant markers between healthy cows and cows with RP. Such findings on potential prognostic markers will reduce the incidence of RP and improve the reproductive and productive performance of dairy cows.

## 2. Materials and methods

### 2.1. Chemicals and materials

All liquid chromatography (LC) solvents [methanol, acetonitrile (ACN), and isopropanol] were of LC-mass spectrometry (MS) grade and purchased from Fisher Scientific (Loughborough, UK). LC-MS additives (formic acid and ammonium acetate) were acquired from Sigma-Aldrich (Madrid, Spain). An ACQUITY UPLC BEH amide column was purchased from Waters (Milford, MA, USA). The vacuum blood collection tubes were purchased from BD Medical Devices Shanghai Co., Ltd. (Shanghai, China). Total glutathione peroxidase (GSH-Px) assay (code no. S0058), malondialdehyde (MDA; code no. S0131S), and total superoxide dismutase (SOD) assay (code no. S0103) detection kits were purchased from Beyotime (Shanghai, China). Endothelin (ET) and estradiol (E_2_) radioimmunoassay (RIA) kits were purchased from Beijing Northern Biotechnology Research Institute Co., Ltd. (Beijing, China). 6-Keto-prostaglandin F1α (6-K) and progesterone (P) enzyme-linked immunosorbent assay (ELISA) kits were purchased from Cayman (Ann Arbor, MI, USA).

### 2.2. Animals and clinical specimens

A single-center, prospective study was conducted in a large-scale pasture from April to November 2021. The annual RP incidence was 14.5% at this large-scale pasture. All experimental procedures involving animals were conducted as per the guidelines of the Animal Care and Use Committee of Ningxia Academy of Agricultural and Forestry Sciences (Yinchuan, China; animal use permit no. 2021-01). The farm in the western part of Yinchuan, Ningxia Hui Autonomous Region has around 5,000 cows, which are fed on a full mixed ration and the herd is tested and monitored for dairy herd improvement (DHI) using an intelligent management system. The main production properties of the herd are as follows: annual milk yield per cow is 9361 ± 661 kg, milk fat percentage is 3.79 ± 0.20, protein percentage is 3.30 ± 0.06 and calving interval is 393.6 ± 5.4 days. 260 Lactating Holstein Friesian dairy cows were included according to the following enrollment criteria: (1) dairy cows had a good physical state and appetite, a body condition score (BCS) between 2.5 and 4.0, and parity between 2 and 3, (2) dairy cows were free of infectious or metabolic diseases, and (3) dairy cows had a clear clinical history of veterinary quarantine and clinical records. Moreover, the dairy cows with a history of cesarean section during the previous or current calving, nutrition-induced prenatal paralysis, subumbilical edema, abortion of large fetus, ketosis and metritis were excluded from the study during the sampling period. Furthermore, dairy cows with severe infections, acute and chronic diseases during the sampling period were also excluded from the study. Blood samples were separately collected from enrolled lactating dairy cows at 21, 14, 7, and 0 days before parturition and at 7, 14, and 21 days after parturition. Blood samples of enrolled cows were obtained from the jugular vein to obtain plasma and/or serum. In brief, blood samples were maintained at room temperature for 1 h and centrifuged at 1,600 *g* for 10 min at 4 °C. The supernatants in the anticoagulation vacuum tubes were transferred into sterile tubes without any preservatives and stored at −80 °C until analysis. The clotted blood in the evacuated tubes without anticoagulant was centrifuged at 2,000 *g* at 4 °C for 20 min, and the supernatant was transferred into sterile tubes. Consequently, blood samples in the transition period from 10 cows with RP were actively collected. To limit the effects of confounding factors on metabolic profiles and clinical laboratory indicators, 10 healthy cows from among the enrolled cows with similar parity (2.4 ± 0.48), BCS (3.2 ± 0.4), and age (3.2 ± 0.51 years) as cows with RP were set as the control group. Moreover, changes in the dry matter intake, milk production, milk composition of healthy cows and cows with RP were detected during the transition periods, and the results are provided in Supplement Materials. No significant changes were noted in the dry matter intake before parturition and milk composition postpartum between the enrolled healthy cows and cows with RP, whereas after parturition, the dry matter intake and milk production were significantly lower cows with RP compared with healthy cows.

### 2.3. Plasma sample preparation

A 100-µL sample was thoroughly mixed with 400 µL cold methanol/ACN (*v*/*v*, 1:1) *via* vortexing. The mixtures were processed by sonication for 1 h in an ice bath. The mixture was incubated at **−**20°C for 1 h and centrifuged at 14,000 *g* at 4°C for 20 min. The supernatants were harvested and dried under vacuum LC-MS analysis. Additionally, to ensure data quality for metabolic profiling, quality control (QC) samples were prepared by pooling aliquots of all representative samples and used for data normalization. QC samples were prepared and analyzed using the same procedure as that used for experimental samples in each batch. Dried extracts were dissolved in 50% ACN. Each sample was filtered using a disposable 0.22 µm cellulose acetate, transferred into 2-mL high-performance LC (HPLC) vials, and stored at −80°C until analysis.

### 2.4. Oxidation and antioxidant markers in serum

The MDA concentrations and the activity of GSH-Px and SOD were assessed through commercial kits following the manufacturer’s protocols.

### 2.5. Reproductive hormone and ET measurement

Serum 6-K and P levels were detected by the corresponding commercial ELISA kits following the manufacturer’s protocols.

Serum E_2_ and ET levels were detected by the corresponding commercial RIA kits. The detailed detection operation was performed by the Beijing North Institute of Biotechnology Co., Ltd.

### 2.6. Ultra-HPLC-tandem MS analysis of plasma metabolites

Metabolomics profiling was analyzed using a UPLC-ESI-Q-Orbitrap-MS system (ultra-HPLC [UHPLC], Shimadzu Nexera X2 LC-30AD, Shimadzu, Japan) coupled with Q-Exactive Plus (Thermo Scientific, San Jose, CA, USA). For hydrophilic interaction LC, samples were analyzed using a 2.1 × 100 mm ACQUIY UPLC BEH amide 1.7 μm column (Waters, Wexford, Ireland). The flow rate was 0.5 mL/min, and the mobile phase contained (A) 25 mM ammonium acetate and 25 mM ammonium hydroxide in water and (B) 100% ACN. The gradient was 95% B for 1 min, linearly reduced to 65% in 7 min, reduced to 35% in 2 min, maintained for 1 min, and increased to 95% in 0.5 min, with a re-equilibration period of 2 min. Electrospray ionization (ESI) positive and negative modes were applied for MS data acquisition. The HESI source conditions were set as follows: spray voltage 3.8 ± 3.2 kV, capillary temperature ±320 °C, sheath gas ±30 arbitrary units, aux gas ±5 arbitrary units, probe heater temperature ±350 °C, and S-lens RF level 50 arbitrary units. In MS-only acquisition, the instrument was set to acquire over the *m/z* range of 80–1,200 Da. The full MS scans were acquired at a resolution of 70,000 at *m/z* 200 and 17,500 at *m/z* 200 for a tandem MS (MS/MS) scan. The maximum injection time was set to 100 and 50 ms for MS and MS/MS, respectively. The isolation window for MS2 was set to 2 *m/z*, and the normalized collision energy (stepped) was set as 27, 29, and 32 for fragmentation.

QC samples were prepared by pooling aliquots of all samples representative of the samples under analysis and used for data normalization. Plasma samples from the two groups were analyzed randomly during the analysis. In addition, QC samples were detected once every five samples for conditioning of the analytical system, signal correction, and quality assurance. To assess the stability of the instruments and systems, the QC sample was run at the beginning, middle and end of the sample queue.

### 2.7. Data preprocessing and filtering

Raw MS data were processed using MS-DIAL (Tsugawa et al. 2020) for peak alignment, retention time correction, and peak area extraction. The metabolites were identified using accuracy mass (mass tolerance < 0.01 Da) and MS/MS data (mass tolerance < 0.02 Da), which were matched with the Human Metabolome Database (HMDB), MassBank, and other public databases and the self-built metabolite standard library (Shanghai Bioprofile Technology Company Ltd). In the extracted-ion features, only variables with >50% of the nonzero measurement values in at least one group were kept. The resulting data table subsequent to data preprocessing was then exported as a .csv file. MetaboAnalyst (Xia et al. 2009) was used to replace missing values in the data table with a half of the minimum value found in the data set and to perform total area normalization (Warrack et al. 2009; Xia et al. 2009). The total area normalization for each sample was performed by dividing the integrated area of each analyte by the sum of all peak areas of analytes present in the sample.

### 2.8. Statistical analysis

#### 2.8.1. Multivariate statistical analysis

R version 4.0.3 and R packages were used for all multivariate data analyses and modeling. Multivariate statistical data analysis was performed on log10-transformed normalized metabolite concentrations to account for the non-normal distribution of the concentration data and reduce the chance of skewed variables. Prior to PCA analysis, multivariate statistical data was mean-centered and unit variance scaled (van den Berg et al. 2006). DModX plot was calculated to check for any outliers. After initial overview of the multivariate statistical data using PCA analysis, the data were subjected to orthogonal partial least-squares discriminant analysis (OPLS-DA) (Bylesjö et al. 2007) wherein a model was built and utilized to identify marker metabolites that enabled healthy cows to be differentiated from cows with RP. OPLS-DA allowed the determination of discriminating metabolites using the variable importance on projection (VIP). The VIP score indicates the contribution of a variable to the discrimination between healthy cows and cows with RP. Mathematically, these scores are calculated for each variable as a weighted sum of squares of PLS weights. The mean VIP value is 1; usually, VIP > 1 is considered significant. A high score agrees with a strong discriminatory ability and thus constitutes the criterion for selecting biomarkers. To investigate model predictability and overfitting of data in the OPLS-DA model, permutation tests with 200 iterations were conducted. These permutation tests compared the goodness of fit of the original model with that of several models based on data where the order of the Y-observations was randomly permuted, while the X-matrix was kept intact (Wiklund et al. 2007; Mahadevan et al. 2008).

The discriminating metabolites were obtained using a statistically significant threshold of VIP values obtained from the OPLS-DA model and a two-tailed Student’s *t*-test (*P* value) on the normalized raw data at the univariate analysis level. *P* values were calculated by one-way analysis of variance for multiple-group analysis. Metabolites with VIP > 1.0 and *p* < 0.05 were considered statistically significant metabolites. Fold change (FC) was calculated as the logarithm of the average mass response (area) ratio between healthy cows and cows with RP. However, the differential metabolites identified were used to perform cluster analyses using the R package (Wu et al. 2021).

To identify the perturbed biological pathways, differential metabolite data were assessed by using the Kyoto Encyclopedia of Genes and Genomes (KEGG) pathway analysis using the KEGG database (http://www.kegg.jp) (Kanehisa et al. 2012). KEGG enrichment analyses were conducted using Fisher’s exact test, and false discovery rate correction for multiple testing was performed. Enriched KEGG pathways were nominally statistically significant at *p* < 0.05.

#### 2.8.2. Correlation analysis of the potential prognostic markers and clinical laboratory indicators

SAS version 9.2 (SAS Institute, Inc., Cary, NC, USA) was used for statistical analysis. The independent-samples *t*-test was used to analyze the differences in clinical laboratory indicators between the analytical groups. Spearman rank correlation analysis was used to explore the relationship between the potential prognostic markers and the clinical laboratory indicators with differences between groups at the same parturition period of dairy cows. Differences with *p* < 0.05 were considered statistically significant.

## 3. Results

### 3.1. Fluctuation of reproductive hormones, ET, and oxidation and antioxidant markers during the transition periods of lactating dairy cows

**Figure 1.**
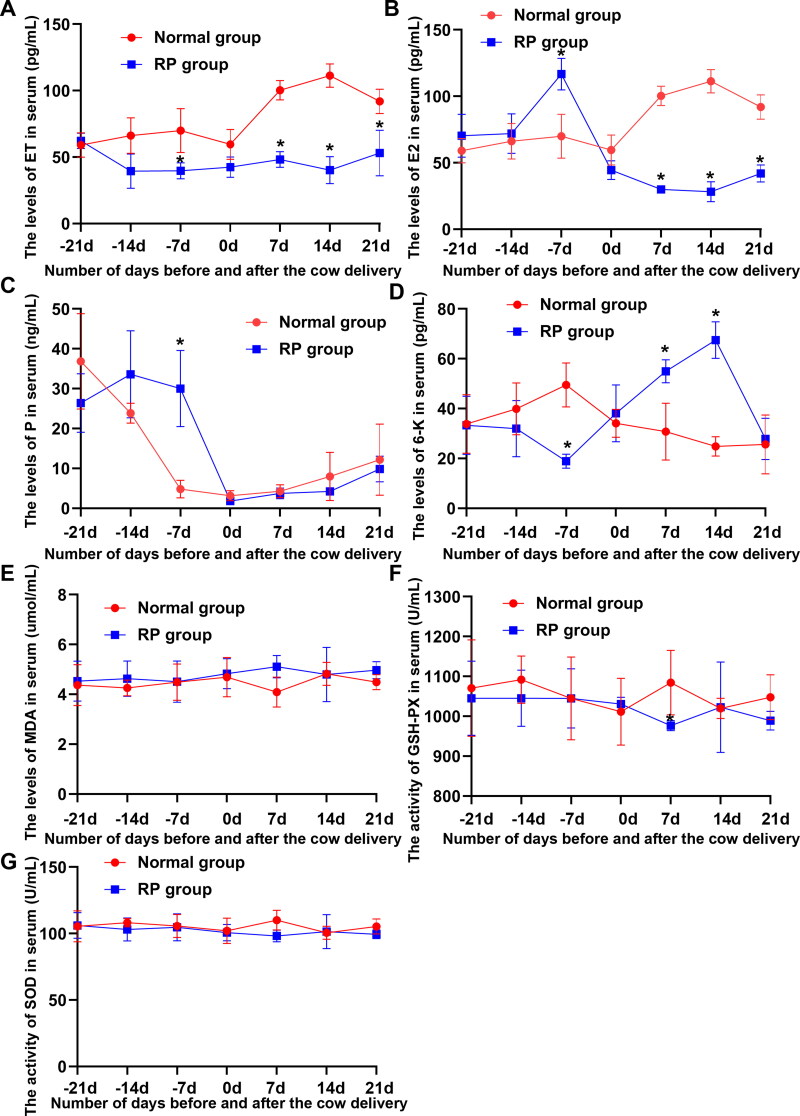
Fluctuation of reproductive hormones, ET, and oxidation and antioxidant markers during the transition periods of lactating dairy cows. (A) Differences in serum ET levels in 10 cows with RP and 10 healthy cows during the transition periods. (B–D) Differences in E_2_, P, and 6-K levels in cows with RP and healthy cows during the transition periods. (E–G) Differences in the MDA level and GSH-Px and SOD activity in cows with RP and healthy cows during the transition periods.

6-K, P and ET play important roles in the biological process of placental maturation and separation. To detemine the fluctuation in 6-K, P and ET during the transition periods, serum 6-K, P, E_2_, and ET levels were assessed. Figure 1A–D shows that 6-K, P, E_2_, and ET levels significantly fluctuated in healthy cows and cows with RP during the transition periods. The serum E_2_ level in dairy cows increased gradually from 21 to 0 days before delivery, whereas, with the advent of parturition, the trend of P changed opposite to that of E_2_. An opposite decreasing trend of 6 K was noted between healthy cows and cows with RP from 21 days before delivery to parturition 14 days.

Oxidative imbalance is considered an important cause of RP in dairy cows. The MDA levels and the activities of GSH-Px and SOD in serum were detected to probe the fluctuation in oxidation and antioxidant markers during the transition periods. No significant decreasing trend was noted in the MDA level and the GSH-Px and SOD activity between healthy cows and cows with RP from 21 days before delivery to day of calving, whereas the MDA level and the GSH-Px and SOD activity significantly fluctuated in cows with RP from parturition to postnatal 21 days (Figure 1E–G).

### 3.2. Reproductive hormones and ET at 7 days before delivery and oxidation and antioxidant markers at postnatal day 7 were significantly different between healthy cows and cows with RP

Differences in reproductive hormones, ET, and oxidation and antioxidant markers between healthy cows and cows with RP in each period were analyzed to screen the period with the most significant difference in physiological indicators between healthy cows and cows with RP. Figure 1A–G shows that ET, 6-K, and E_2_ levels in the serum of healthy cows were significantly higher than those in the serum of cows with RP at 7 days before delivery, whereas the P level was significantly reduced. The SOD and GSH-Px activities of healthy cows at postnatal day 7 were significantly higher than those of cows with RP, and the MDA level in healthy cows was significantly reduced.

### 3.3. Differential metabolic profile between healthy cows and cows with RP at 7 days before delivery

**Figure 2.**
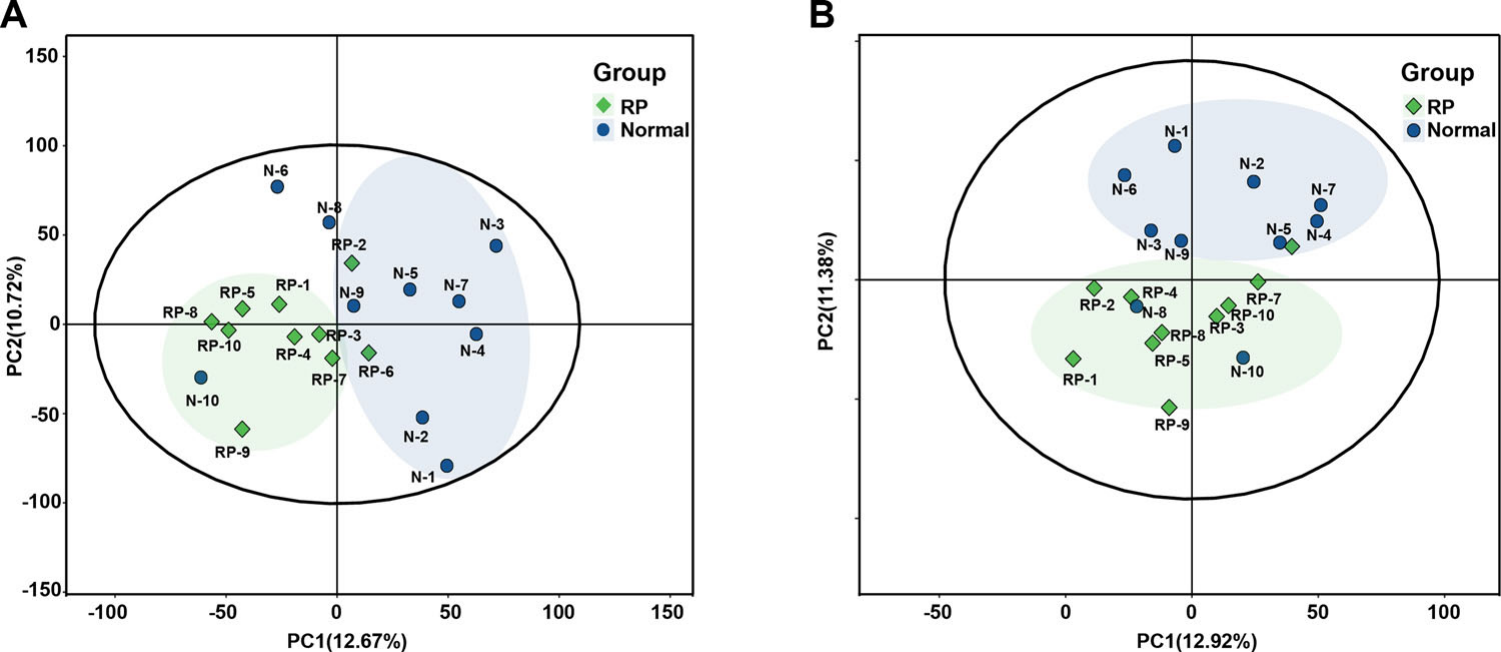
Differential metabolic profiles of plasma of 10 healthy cows and 10 cows with RP. (A and B) PCA score plots based on plasma metabolic profiles of healthy cows and cows with RP in positive and negative modes. *ESI+*: *R*^2^ = 0.568, *ESI−*: *R*^2^ = 0.603.

As aforementioned, reproductive hormones and ET showed the most significant difference at 7 days before delivery between healthy cows and cows with RP, and the metabolic profile of healthy cows and cows with RP at 7 days before delivery as detected by UHPLC-MS/MS. Moreover, 483 and 358 metabolites were identified based on metabolite ion peaks in positive and negative ion modes, respectively. Figure 2A and B shows that the sample points between groups showed a trend of separation in the generated PCA score plots and the sample points within a group showed a trend of convergence in positive and negative modes. These findings suggested a differential metabolic profile between healthy cows and cows with RP at 7 days before delivery of the neonate.

### 3.4. Potential biomarkers between healthy cows and cows with RP referred to multiple biological pathways involved in organic acids and derivatives

**Figure 3.**
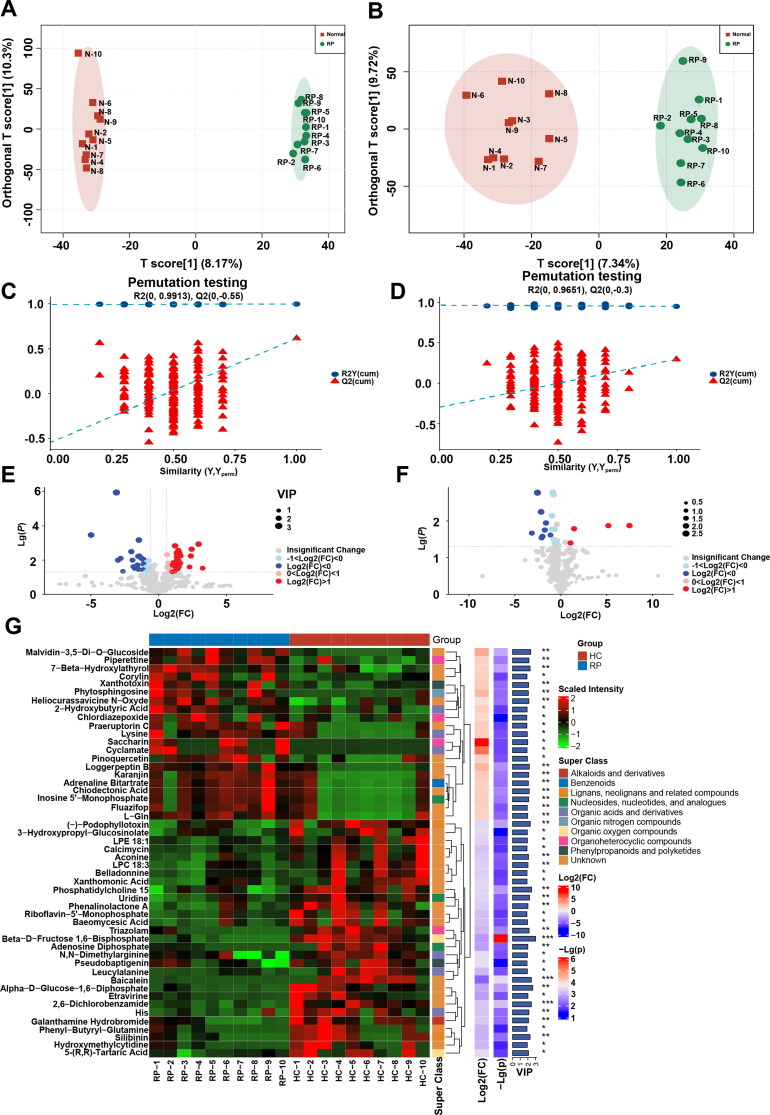
Screening of differential metabolites in plasma of 10 healthy cows and 10 cows with RP. (A and B) OPLS-DA score plots based on serum metabolic profiles of healthy cows and cows with RP in positive and negative modes. (C and D) Permutation test of OPLS-DA model for different comparison groups. (E and F) Volcano plots based on VIP, *P* value, and FC of differential metabolites of healthy cows and cows with RP in positive and negative modes. (G) Differential metabolite hierarchical clustering results based on VIP, *P* value, FC, and superclass of differential metabolites of healthy cows and cows with RP.

**Figure 4.**
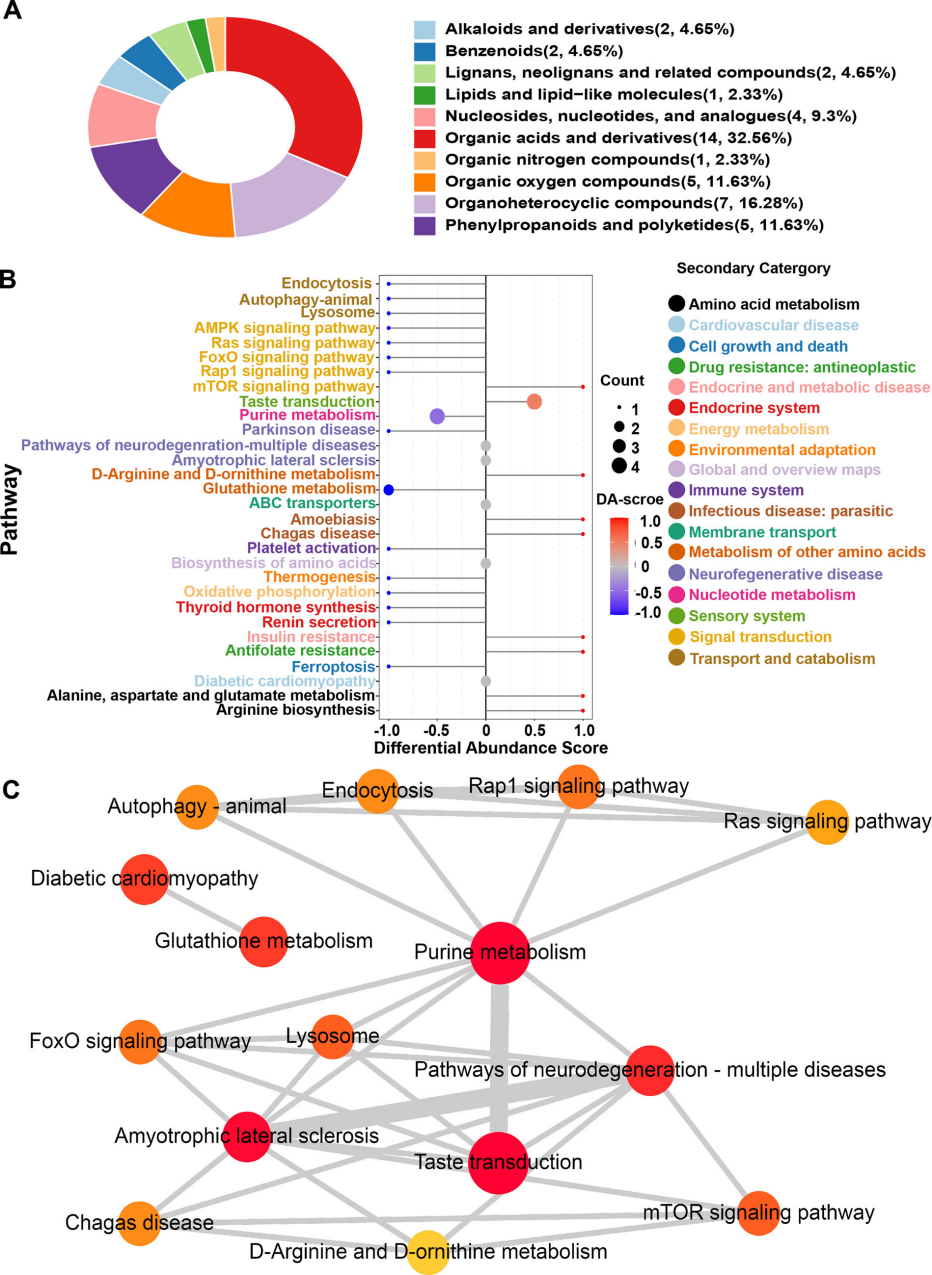
Taxonomic, functional, and pathway analyses of differential metabolites. (A) Differential metabolite classification ring diagram based on matching results against the HMDB database. (B) Differential abundance scores (DA score) shows a trend of overall downregulation of metabolic pathways. **C** Network map of differential metabolite enriched pathways. Each point represents a pathway. The *color* represents the significant *P* value of enrichment. The redder the point represents, the more significant the enrichment of the pathway. The *size of the circle* represents the number of metabolites annotated to the pathway (count), and the larger the circle is, the higher the count. Network connections were noted between the pathways, indicating that they contain common differential metabolites; the thicker the line is, the more metabolites are shared by the two pathways.

OPLS-DA analysis was performed to further screen for significant differential metabolites (Figure 3A–D). Based on VIP > 1 obtained using the OPLS-DA model and *p* < 0.05 and FC > 1.5 or FC < 0.667 obtained using univariate statistical analysis, differential metabolites with biological significance were further mined. In total, 74 and 21 differential metabolites were mined in positive and negative ion modes, respectively. A volcano map and cluster heatmap analyses were performed to visualize the analysis of potential biomarkers and expression patterns of potential biomarkers within different samples (Figure 3E–G). The expression patterns of potential biomarkers were significantly different in samples between groups with similar expression in samples within groups (Figure 3G). Based on the structure and function of the metabolites, the potential biomarkers in each comparison group were classified and counted by comparing them to the HMDB database. These potential biomarkers were divided into several categories, including alkaloids and derivatives, benzenoids, lignans, neolignans and related compounds, nucleosides, nucleotides, and their analogs, organic acids and derivatives, organic nitrogen compounds, organic oxygen compounds, organoheterocyclic compounds, phenylpropanoids and polyketides, and others (Figure 4A). Organic acids and derivatives accounted for 32.5% of the annotated differential metabolites (Figure 4A).

KEGG ID mapping was performed on differential metabolites in the comparison groups to clarify the function of metabolites and the relationship between metabolites. Figure 4B shows that the potential biomarkers were involved in the mammalian target of rapamycin (mTOR) signaling pathway, purine metabolism, taste transduction, and other biological processes. The functional interactions of these pathways were analyzed by Cytoscape to further understand the functional interactions of pathways enriched by potential biomarkers. Figure 4C shows that the purine metabolism pathway was a hub for other related pathways.

**Figure 5.**
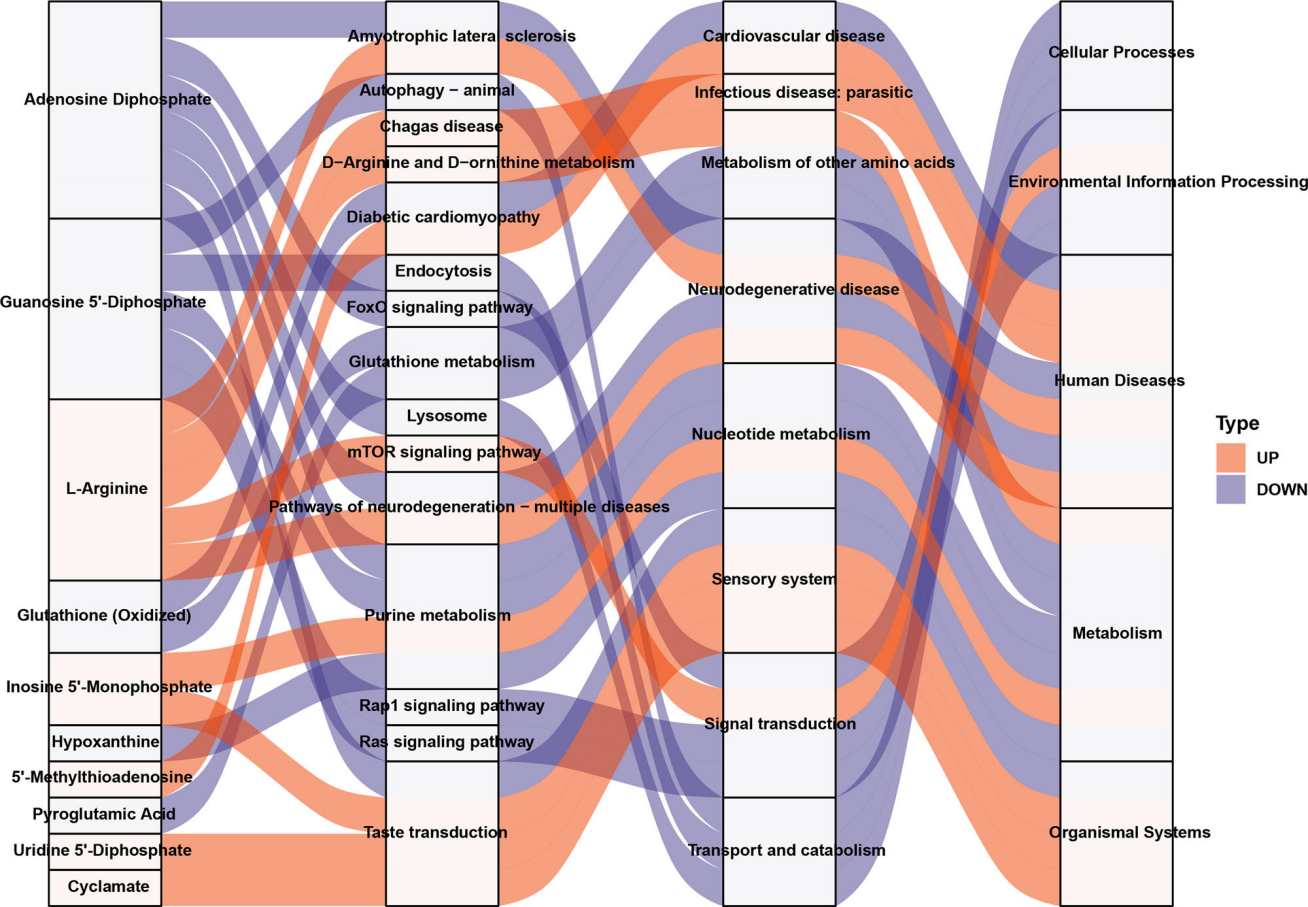
The Sankey diagram showing data flow analysis of upregulated and downregulated metabolites and KEGG pathway level. The *first column on the left* indicates the up and down differential metabolites. The *height of the box* indicates the amount of data flow, i.e. the number of metabolites annotated to the pathway. The more the corresponding pathway is, the higher the box. The *red streamline* indicates the upregulated (up) metabolite, and the *flow direction of the blue streamline* indicates the flow direction of downregulated (down) metabolites. The *second, third, and fourth columns* indicate the pathway, and the level increases accordingly.

A Sankey energy balance diagram was drawn to visualize the data flow of potential biomarkers and the KEGG pathway level. Figure 5 shows 10 potential biomarkers (Table 1) involved in the top 15 differential enriched pathways.

**Table 1.** Relative abundance of significant plasma metabolites (mean and SEM) in healthy control (CON; *n* = 10) and retained placenta (RP; *n* = 10) cows Result of biomarkers identified in the plasma of cows with RP.

Metabolite(IPRI)	RP^a^	SEM	CON^b^	SEM	*P*-value	FC (T/C)
Adenosine Diphosphate*	1.51E + 06	6.14E + 05	1.06E + 07	2.98E + 06	0.008	0.14
Guanosine 5′-Diphosphate*	1.49E + 05	3.73E + 04	1.33E + 06	4.68E + 05	0.021	0.11
L-arginine*	1.12E + 10	1.19E + 09	5.55E + 09	1.86E + 09	0.020	2.01
Glutathione (oxidized)*	1.09E + 06	1.73E + 05	4.31E + 06	1.32E + 06	0.026	0.25
Inosine 5′-Monophosphate*	4.29E + 08	6.20E + 07	1.50E + 08	5.69E + 07	0.004	2.86
Hypoxanthine*	1.54E + 08	2.07E + 07	2.25E + 08	2.20E + 07	0.031	0.69
D-glucosamine-6-phosphate*	2.88E + 08	6.24E + 07	1.44E + 08	2.75E + 07	0.048	2.00
Pyroglutamic acid*	9.12E + 08	4.44E + 07	1.12E + 09	6.80E + 07	0.020	0.71
Saccharin*	1.83E + 09	6.64E + 08	1.03E + 07	6.20E + 06	0.013	177.69
Cyclamate*	1.03E + 09	3.66E + 08	2.87E + 07	8.36E + 06	0.013	35.98

^a^Cows with RP. ^b^Healthy cows. *p < 0.05 compared to healthy cows; T/C cows with RP compared to healthy cows. IPRI: ion peak relative intensity.

### 3.5. Analysis of sensitivity and specificity of prediagnostic biomarkers

**Figure 6.**
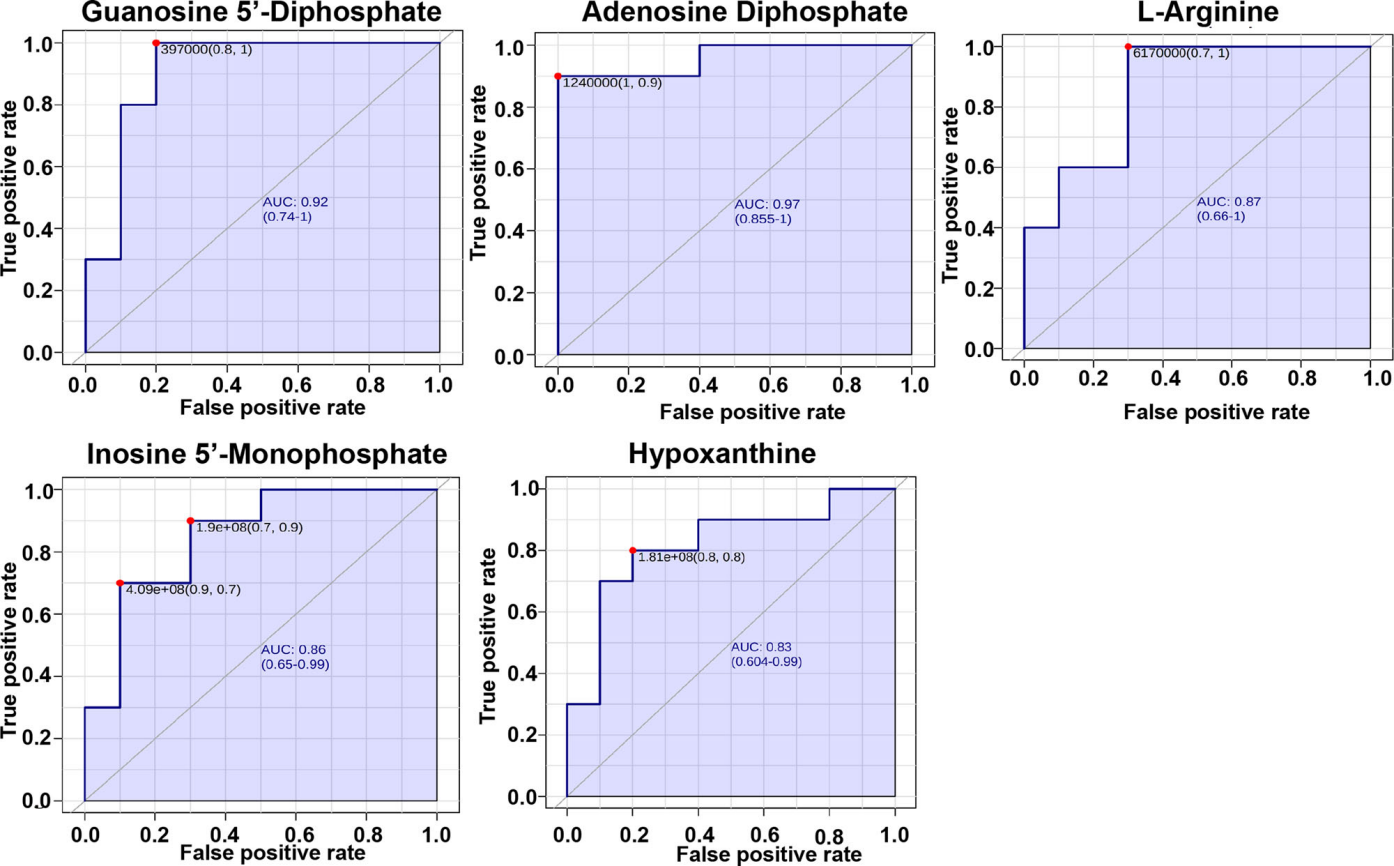
The receiver-operator characteristic curves of Adenosine Diphosphate、Hypoxanthine, Guanosine 5'-Diphosphate, Inosine 5'-Monophosphate, L-arginine that accounted for the differentiation of 10 healthy cows and 10 cows with RP.

The receiver operating characteristic (ROC) curve analysis of 10 potential biomarkers was applied to further optimize the high sensitivity and specificity of prediagnostic biomarkers. Figure 6 shows that adenine dinucleotide phosphate (ADP), hypoxanthine (hyp), guanine dinucleotide phosphate (GDP), inosine monophosphate (IMP), and L-arginine were used as potential biomarkers with good ability to distinguish healthy cows from cows with RP. Moreover, the combination of ADP, GDP, hyp and IMP in the prediagnosis of RP is more sensitive and specific (Figure S1).

### 3.6. Close relationship of prediagnostic biomarkers with reproductive hormones and ET

**Figure 7.**
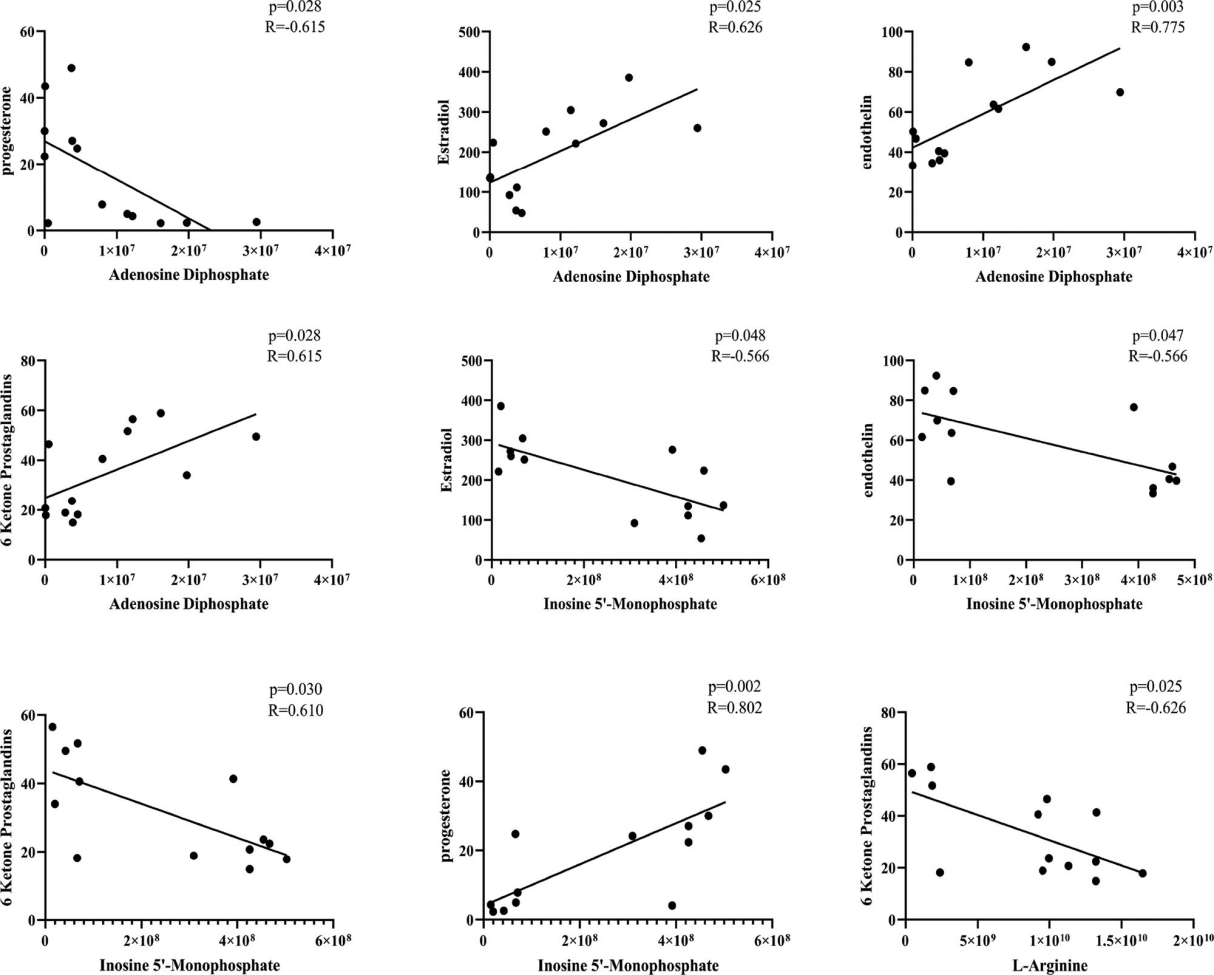
Correlation analysis of differential metabolites and related reproductive hormones that accounted for the differentiation of 10 healthy cows and 10 cows with RP.

A correlation analysis of prediagnostic biomarkers and the reproductive hormones and ET was conducted to investigate the relationship of prediagnostic biomarkers with reproductive hormones and ET. Figure 7 shows that ADP and hyp were positively correlated with ET (*p* = 0.013, *R* = 0.621 and *p* = 0.009, *R* = 0.651), E_2_ (*p* = 0.015, *R* = 0.612 and *p* = 0.031, *R* = 0.557), and 6-K (*p* = 0.022, *R* = 0.583 and *p* = 0.002, *R* = 0.721). Meanwhile, ADP and hyp showed a negative correlation with P (*p* = 0.032, *R* = −0.555 and *p* = 0.028, *R* = 0.564), respectively. L-arginine showed a negative correlation with 6-K (*p* = 0.013, *R* = −0.623). IMP showed a negative correlation with ET (*p* = 0.009, *R* = −0.648), E_2_ (*p* = 0.027, *R* = −0.569), and 6-K (*p* = 0.017, *R* = −0.603). Meanwhile, IMP was positively correlated with P (*p* = 0.01, *R* = 0.644). These findings suggested that ADP, L-arginine, and IMP had a close relationship with reproductive hormones and ET.

## 4. Discussion

At present, the incidence of RP morbidity varies across countries and herds, depending on the management and environment of herds; the physiological state of cows including age, parity, heredity, hormones, and nutrition and the condition of the calves, such as stillbirth and twinning (Han and Kim 2005; Mahnani et al. 2021b). To limit the effects of confounding factors on metabolic profiles and clinical laboratory indicators, 10 healthy cows from enrolled cows with a similar parity, BCS, and age as cows with RP were set as the control group. Moreover, before parturition there were no significant changes in dry matter intake and milk composition postpartum between enrolled the healthy cows and cows with RP. Therefore, parity, age and BCS were not set as covariates in the subsequent metabolite differential analysis. Placental maturation and separation in dairy cows is a complex physiological process regulated by reproductive hormones, cytokines, and other factors (Attupuram et al. 2016). Moreover, these regulators fluctuate with the progression of placental maturation and separation. Consistent with previous reports (Takagi et al. 2002; Garcia-Ispierto et al. 2016; Molefe and Mwanza 2020), this study showed that 6-K, P, E_2_, and ET levels significantly fluctuated during the transition periods of healthy cows and cows with RP. The MDA level and the GSH-Px and SOD activity significantly fluctuated in cows with RP from delivery to postnatal 21 days. The dysregulation of reproductive hormones and ET is an important incentive to hinder placental maturation and separation (Uyanikoglu et al. 2018). In this study, reproductive hormones and ET were most significantly different between healthy cows and cows with RP at 7 days before delivery, demonstrating the close relationship between dysregulation of ET and reproductive hormones and RP in dairy cows. Serum ET, 6-K, and E_2_ levels in healthy cows were significantly higher than those in cows with RP at 7 days before delivery, whereas P levels were significantly reduced. ET was involved in a series of biological effects, including ovarian steroid hormone secretion, luteal degeneration, early placenta formation and fetal delivery in the reproductive system (Takagi et al. 2002). ET could act on the vascular tissue on the surface of the chorion or placenta to regulate its contraction, thereby affecting the metabolism of the placenta (Beagley et al. 2010; Uyanikoglu et al. 2018). A highly coordinated action of ET, E_2_, PGF2 and P is required for placental formation, maturation, and separation. In placental maturation and separation, ET, E_2_, and PGF levels were synergistically elevated and contributed to the inhibition of P secretion by activating ET receptors (Takagi et al. 2002). Elevated P levels can inhibit uterine contractions and reduce vasopressin secretion through negative feedback (Takagi et al. 2002). The SOD and GSH-Px activities of healthy cows at day 7 postnatal were significantly higher than those of cows with RP, and the MDA level in healthy cows was significantly reduced. This trend of increasing MDA and decreasing GSH-Px and SOD activities in the serum of postnatal cows with RP has been documented. The significant increase in oxidative markers of cows with RP at postnatal day 7 may be due to oxidative stress caused by RP. Oxidative stress is considered a result or a cause involved in placenta expulsion (Menon and Richardson 2017).

Choosing an appropriate diagnosis period is necessary to improve the accuracy and sensitivity of prediagnostic indicators. Several physiological and biochemical indicators were not consistently and significantly altered at different detection periods. Consistent with previous reports, in this study, the best prediagnosis period was 7 days before delivery with the most significant difference in ET and reproductive hormones between healthy cows and cows with RP.

The metabolism of dairy cows undergoes an immense adaptive change from late pregnancy to the early postpartum period (Dervishi et al. 2016; Dervishi et al. 2018; Lu et al. 2020a). Dairy cows can experience a negative energy balance in early lactation when feed intake is too low to meet the energy requirements for body maintenance and milk production. Metabolic changes occur in animals experiencing a negative energy balance (Xu et al. 2020). The incidence of reproductive diseases, e.g. abortion and RP, was closely associated with the emerging metabolic stress. Many metabolomics approaches have been applied for the purpose of differential phenotyping and biomarker identification of diarrhea and RP in dairy cows because the metabolic phenotype is the most sensitive response to physiological or pathological changes in the organism. In this study, the results of differential metabolic profiles between healthy cows and cows with RP at 7 days before delivery further confirmed that the physiological and biochemical characteristics of cows in the transition period had undergone considerable changes before delivery. Moreover, 95 potential biomarkers involved in the mTOR signaling pathway, purine metabolism, taste transduction, and adenosine deaminase (ADA) deficiency disease pathway were mined, and organic acids and derivatives accounted for 32.5% of the annotated differential metabolites. Five potential biomarkers, including ADP, hyp, GDP, IMP, and L-arginine, were screened as prediagnostic biomarkers by functional pathway enrichment, ROC, and correlation analyses to further optimize the high sensitivity and specificity of prediagnostic biomarkers. This discussion will focus on changes in these potential prediagnostic biomarkers.

ATP and ADP play an important role in the maturation and separation of the placenta as important energy molecules (Dervishi et al. 2016). Insufficient energy supply will lead to the retardation of placental material transport and the transmission of biological information, resulting in the blockage of placental development, maturation, and separation (Dervishi et al. 2016; Martinez et al. 2016). Many ATP- or ADP-dependent enzymes were noted in the placenta, e.g. ATP-dependent protease, which played a vital role in the regulation of steroid hormone metabolism (Thomas et al. 2019). In this study, ADP levels significantly decreased in cows with RP. Decreased ADP levels in dairy cows would disrupt placental material metabolism and related regulatory signal transduction. In this study, compared with healthy cows, the hyp and GDP levels in cows with RP were significantly decreased and IMP levels in cows with RP were significantly increased. The metabolism of these purine compounds, including hyp, GDP, and IMP, was regulated by multiple signaling pathways. ADA could regulate the purine salvage pathway to affect purine metabolism (Jadhav and Jain 2013). ADA is highly expressed on the placenta and chorion during fetal stages of development, which is essential for fetal development and placenta maturation (Jadhav and Jain 2013). ADA deficiency would cause the deregulation of purine metabolism and ADA substrate accumulation. In this study, purine metabolites, e.g. hyp, GDP, and IMP, were severely disordered. Thus, purine metabolic disturbances may be associated with ADA disorders. Moreover, the pathway enrichment results of the differential metabolites also suggested that these differential metabolites may be involved in ADA deficiency diseases and autophagy. Autophagy was involved in the entire process of placental maturation and separation (Brickle et al. 2015). Once the balance of autophagy is disrupted, it will lead to placental dysplasia and abscission (Oh and Roh 2017; Sotthibundhu et al. 2021).

However, the role of ADA in cows with RP and in the regulation of the purine metabolism need to be further explored. Arginine plays a vital role in protein and pyrimidine synthesis and the release of various hormones. In this study, L-arginine levels significantly decreased in cows with RP, but the cause for elevated L-arginine was still unclear.

In summary, 7 days before parturition is the best period for the early-warning diagnosis of RP in dairy cows. Increasing L-arginine and IMP levels and decreasing ADP, GDP and hyp levels were identified in the plasma of cows with RP, and purine metabolism and autophagy may play a vital role in the occurrence and development of RP in dairy cows. The findings of potential prognostic markers might reduce the incidence of RP and improve the reproductive and productive performance of dairy cows.

## Supplementary Material

Supplemental Material

## Data Availability

The authors confirm that the data supporting the findings of this study are available within the article [and/or] its supplementary materials.
