# Supplementary material for: Potential prognostic markers of retained placenta in dairy cows identified by plasma metabolomics coupled with clinical laboratory indicators
Source: Vet Q. 2022 Nov 13;42(1):199–212. doi: 10.1080/01652176.2022.2145619 (PMC9668283; doi:10.1080/01652176.2022.2145619)
Supplement: Supplemental Material [file TVEQ_A_2145619_SM6591.zip › supplyment materials/Figure S1.pdf]

The gamboost (Boosted Generalized Linear Model) method was used to model the four metabolites (ADP, GDP, Hyp and IMP by the LOOCV (Leave one out cross-validation) validation method. The ROC plot of the gamboost mode as followed:

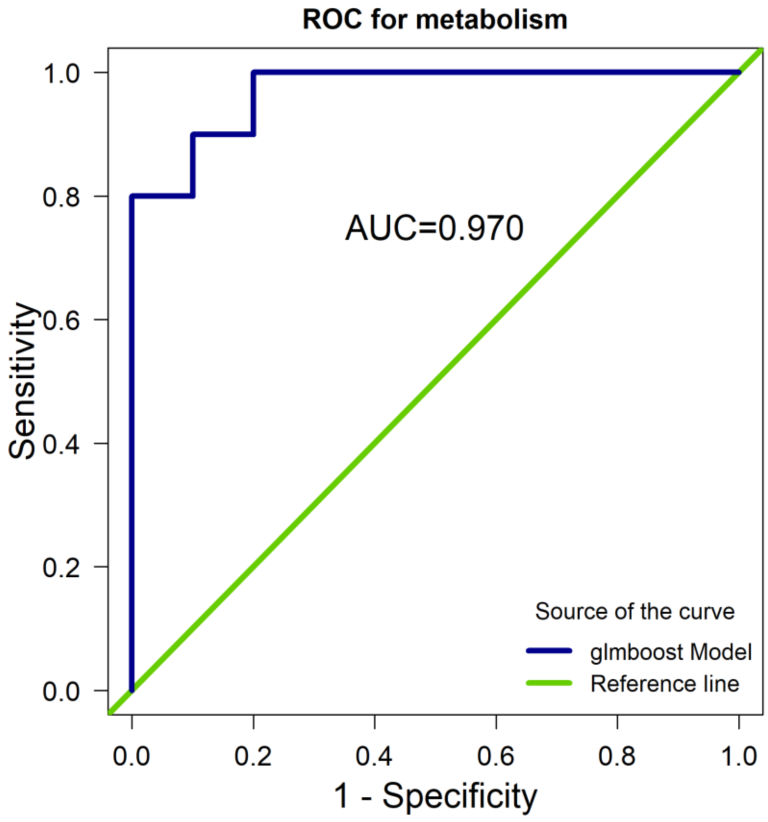

Figure s1 ROC plot of the gamboost model
