# Supplementary material for: Potential prognostic markers of retained placenta in dairy cows identified by plasma metabolomics coupled with clinical laboratory indicators
Source: Vet Q. 2022 Nov 13;42(1):199–212. doi: 10.1080/01652176.2022.2145619 (PMC9668283; doi:10.1080/01652176.2022.2145619)
Supplement: Supplemental Material [file TVEQ_A_2145619_SM6591.zip › supplyment materials/supplyment materials.pdf]

Data of dry matter intake (DMI), milk composition and milk production of dairy cows with (n=10) and without (n=10) retained during the periparturient period (Mean±SD).

**Group**	**Healthy cows**							**Cows with RP**						
**Days relative to parturition (d)**	-21	-14	-7	0	7	14	21	-21	-14	-7	0	7	14	21
**DMI, Kg/d**	14.1±0.19	13.5±0.21	12.6±0.17	12.1±0.13	16.9±0.23	16.8±0.11	18.5±0.27	13.8±0.24	13.5±0.29	12.4±0.19	12.2±0.07*	15.6±0.12*	16.3±0.09*	17.8±0.14*
**Milk production, Kg/d**	-	-	-	22.8±0.42	33.5±0.54	33.7±0.25	38.06±0.91	-	-	-	20.6±0.37*	26.2±0.49*	27.7±0.52*	31.2±0.79*
**Fat ratio(%)**	-	-	-	4.3±0.13	3.8±0.22	3.7±0.09	3.6±0.14	-	-	-	4.2±0.08	3.8±0.21	3.6±0.12	3.6±0.16
**Protein ratio(%)**	-	-	-	3.4±0.08	3.3±0.04	3.3±0.07	3.2±0.04	-	-	-	3.4±0.06	3.3±0.07	3.3±0.03	3.3±0.06

*P < 0.05 compared to healthy cows at the same days relative to parturition; “-“ : The indicator was not detected during this period
